# The Italian Wall Lizard *Podarcis siculus* as a Biological Model for Research in Male Reproductive Toxicology

**DOI:** 10.3390/ijms232315220

**Published:** 2022-12-02

**Authors:** Luigi Rosati, Teresa Chianese, Palma Simoniello, Chiara Maria Motta, Rosaria Scudiero

**Affiliations:** 1 Department of Biology, University Federico II, Via Cintia 21, 80126 Napoli, Italy; 2 Department of Sciences and Technology, University Parthenope, Centro Direzionale, Isola C4, 80143 Napoli, Italy

**Keywords:** endocrine disrupting compounds, environmental contaminants, reptiles, spermatogenesis, testis

## Abstract

Spermatogenesis is a genetically driven differentiation process that occurs in the testis and leads to the formation of spermatozoa. This process is extensively studied in several experimental models, particularly in vertebrates that share the morphological structure and functionality of the mammalian testis. Although reptiles are not generally considered biological models, the lizard *Podarcis siculus* has represented a suitable organism for the study of spermatogenesis over the years. In this lizard, the process of spermatogenesis is regulated by the interaction between systemic factors such as gonadotropins and local factors, i.e., molecules produced by the somatic and germinal cells of the testis. Many exogenous substances are able to alter the production of these regulative factors, thus altering the course of spermatogenesis, and *P. siculus* has proven to be an excellent model for studying the effects of various endogenous or exogenous substances on mechanisms underlying spermatogenesis. This review summarizes the available data on the effects of different substances on the control of spermatogenesis, highlighting the induced morphological and molecular alterations. Overall, the data show that sex hormone levels as well as the final stages of spermatogenesis are most affected by an imbalance of endogenous compounds or contamination by environmental pollutants. This is helpful for the male individual, since the damage, not affecting the spermatogonial stem cells, can be considered transient and not irreversible.

## 1. Introduction

**Figure 1:**
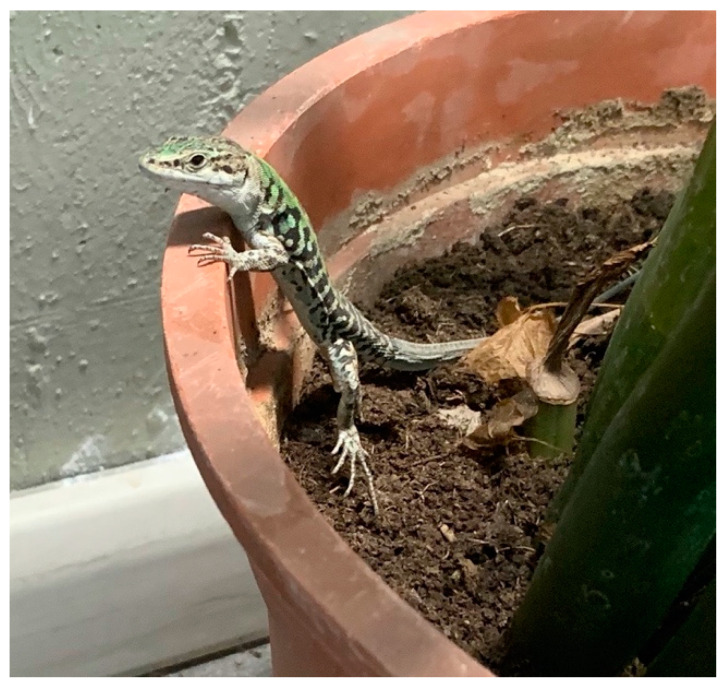
Male specimen of *Podarcis siculus* in an antrophic environment.

Among the reptiles inhabiting the Italian peninsula, the specimens of the field lizard *Podarcis siculus* (Rafinesque-Schmaltz, 1810) (Sauria, Lacertidae) are the most abundant (Figure 1). The Italian wall lizard, formerly known as *Lacerta sicula*, then *Podarcis sicula*, and currently *P. siculus*, is an endemic species of the Mediterranean regions, from the Iberian Peninsula to Tunisia [1]. It prefers warm, arid climates and for this it tends to disappear over 1000 m altitude. Their habitat varies from rural areas to cultivated fields and city gardens. It is often considered an invasive alien species dangerous to the native species [2]; a population had even been introduced in 1967 to New York [3], and, more recently, an abundant population has been found in a Natural Ornithological Park in Russia [4]. Adult specimens reach a length of 25 cm and a weight of 15 g; males are larger, have larger heads, and longer hind limbs. Sexual dimorphism becomes particularly evident during the reproductive season, when males develop femoral pores and aggressive behavior. They feed on larvae and adult insects, worms, and, occasionally, fruits and vegetables; several cases of cannibalism have been reported [5].

Its presence close to urban centers and the ability to live in altered anthropogenic habitats, without a significant loss of biodiversity over time, allows it to be considered a non-threatened IUCN species [6]. In addition, the possibility of keeping animals even for a long time in terrariums has made this lizard an interesting experimental model to study the biology of Reptilia in general, and reproduction in particular, in a typical oviparous species. In captivity, *P. siculus* specimens in fact continue to exhibit the essential features shown in the wild provided that natural photothermal conditions are maintained. Reptiles are pivotal in vertebrate evolution and were the first to develop the amniote condition. Although they are an often-neglected biological model, they can provide interesting information on comparative endocrinology and, above all, on developmental biology.

Studies on *P. siculus* gametogenesis and embryonic development have accumulated since the 1960s. The advancement of morphological and molecular techniques has provided increasing details on mechanisms of neuroendocrine control, on hormones, and on hormone receptors; many studies have also concerned the processes of vitellogenesis and oocyte growth.

In more recent years, *P. siculus* has been used also in toxicity studies providing evidence on responses to soil contamination; in fact, this lizard lives in strict contact with the soil, and contaminants can be absorbed via skin, inhalation, or diet [7]. Eggs are laid in soil, and since they have a water-semipermeable shell, they can absorb contaminants such as pesticides or pollutants dispersed by contaminated superficial water used for irrigation [8,9,10]. For this reason, toxicological studies have increased considerably in recent years in the attempt to determine the impact of environmental pollution on reproductive fitness.

This review summarizes the research carried out over more than a half-century and illustrates the great contribution provided to reproductive biology and, in particular, to reproductive toxicology.

## 2. *Podarcis siculus* Reproductive Cycle

Gametogenesis is a sophisticated and complex morphogenetic process that takes place in the gonads, where the differentiation of gonia into mature gametes occurs under the control of both systemic and local molecules.

In eterothermal species living in temperate areas, such as *P. siculus*, gametogenesis is under the control of the hypothalamus–hypophysis–gonad (HHG) axis, which in turn is controlled by the photothermal regime. In *P. siculus*, mating and ovulation are confined to a brief period every year. Between late April and mid-July, females lay 12 to 20 eggs in 2–3 episodes about 20 days apart [11]. Clutch size depends on female size and resources. Eggs are buried by the mother in shallow holes that are partially guarded by the mother. Seasonality assures that youngs emerge in summer, the time when environmental factors assure optimal temperature and food supply (ultimate factors).

The initiation and modulation of reproductive processes, however, are timed earlier, by predictive proximate factors represented by seasonal changes in day length. In *P. siculus,* in fact, gametes development takes a year to be completed. In females, follicles are recruited in fall, remain quiescent in winter, and start vitellogenesis in early spring [12]. In the male, spermatogenesis begins in fall, when only a few sperm are produced and not used. They complete differentiation in later early spring. After mating, the animals enter a refractory status [13], leading to a condition of physiological hypophysectomy, during which they are unresponsive to endogenous or exogenous hormones or temperature manipulation.

Seasonal changes in day length, therefore, act as a zeitgeber entraining the endogenous circannual rhythm [14,15]. *P. siculus* is believed to possess an endogenous reproductive rhythm, by virtue of the observations that populations belonging to the same species, despite experiencing different conditions of daylight and ambient temperature at different latitudes, breed in the same period of the year [16]. It has been demonstrated that *P. siculus* males kept in constant darkness at 5 °C or in daily photophases of 14 h at 28–30 °C from late summer onwards showed a testicular recrudescence in the following March at about the same period of the naturally occurring event [16].

## 3. *Podarcis siculus* Spermatogenesis

### 3.1. Testis Morphology

**Figure 2:**
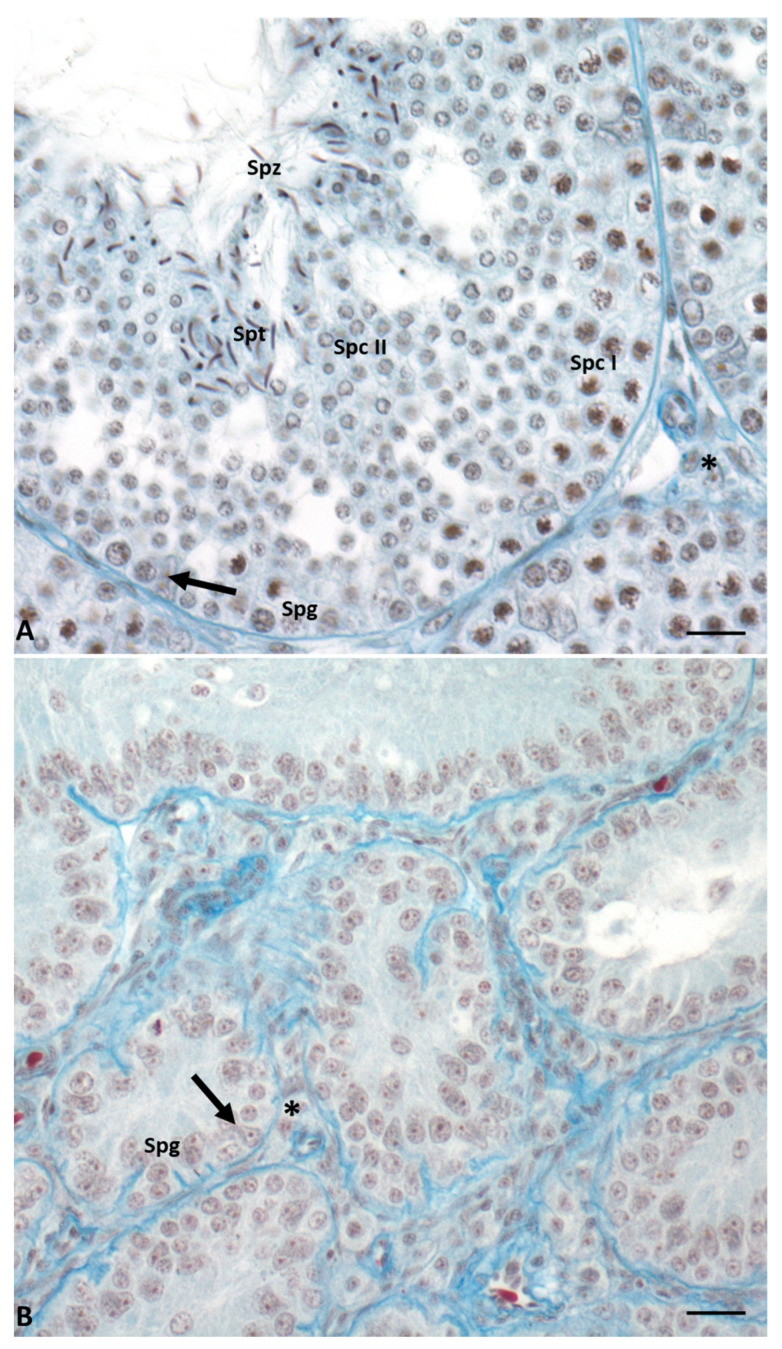
Cross-section of control lizard testis, stained with Mallory’s trichrome, during the two main phases of the reproductive cycle: reproductive period (**A**) and summer stasis (**B**). (**A**) During this period, the seminiferous tubules are characterized by a large lumen and a thick wall with germ cells in all stages of differentiation, i.e., spermatogonia (Spg), spermatocytes I (Spc I) and II (Spc II), spermatids (Spt), and spermatozoa (Spz). Triangular-shaped Sertoli cells (arrow) and Leydig cells in the interstitial space (asterisk) are clearly visible. (**B**) In this period, seminiferous tubules are smaller and characterized by the absence of lumen and the presence of only spermatogonia (Spg) in the thin wall. Sertoli cells (arrow) and Leydig cells in the interstitial space (asterisk) are always present. Scale bars at 10 µm.

In this lizard, testes have a typical tubular organization that changes during the annual cycle determining six different conditions [17,18,19,20]. During the breeding season (May–June), the seminiferous epithelium is thick, germ cells are in all stages of differentiation, and it is rich in spermatozoa ready to be ejaculated. In the summer stasis (July–August), tubules regress and the epithelium becomes thin and is composed only of spermatogonia and Sertoli cells. In early (September) and mid-autumn (October–November), a recovery occurs: spermatogenesis is resumed, spermatocytes I (early autumn) are produced, and then all the germ cells, including few spermatozoa (mid-autumn). The latter, however, are not used for reproduction; this spermatogenic recovery is considered the reminiscence of two reproductive events once present in the ancestor of this lizard, living in a milder environment [20]. This morphological organization of the seminiferous tubules is maintained during the winter stasis, while during spring recovery there is the complete activation of spermatogenesis, and the tubules are rich in spermatozoa ready to be ejaculated (Figure 2). Photoperiod and temperature are the most important players in the regulation of spermatogenesis [15,19,21,22].

### 3.2. Control of Spermatogenesis

*P. siculus* represents one of the main animal models used to study the intricate process of spermatogenesis since the 1950s, as evidenced by the studies of Galgano and D’Amore [17]. Over time, an abundance of studies has documented spermatogenesis control mechanisms in *P. siculus*, many of which helped to elucidate the spermatogenic process in other vertebrates that share a similar testicular organization, including mammals. It has been shown that the process is regulated by a network of interaction between several factors of both systemic and testicular origin [20]; among these, the pituitary adenylate cyclase activating polypeptide (PACAP) and vasoactive intestinal peptide (VIP) [23,24,25,26,27,28], D-aspartic amino acid (D-Asp) [29,30], beta-endorphin [31,32], retinoic acid [33], and steroidogenic enzymes [27,34,35] deserve to be mentioned. Numerous studies have demonstrated the presence in *P. siculus* testis of these molecules with their corresponding receptors both in the germline and in somatic cells, suggesting their involvement in the control of testicular activities [24,25,26]. In detail, qPCR experiments revealed a cyclical pattern of *PACAP* and *VIP* mRNA levels during the different phases of the reproductive cycle, with the lowest mRNA expression levels recorded during the summer and winter stasis, when spermatogenesis is blocked; the highest transcript levels were detected during the reproductive period, characterized by maximum testicular activity; finally, intermediate expression levels were found in the recovery phases, when spermatogenesis is reactivated [24,25,26]. These data, therefore, highlight that the seasonal changes in *PACAP* and *VIP* mRNA levels are directly linked to changes in *P. siculus* testis activity. Furthermore, in situ hybridization and immunocytochemical investigations have shown that PACAP, VIP, and their receptors are widely distributed in the Podarcis testis throughout the reproductive cycle and localized in both germ cells and somatic cells [24,25,26,27,28,34]. Taken together, all these data suggest that PACAP and VIP could act as local control factors of spermatogenesis and spermiogenesis in *P. siculus*.

In addition to neuropeptides, the presence in the testis of *P. siculus* of beta-endorphin (β-EP), an endogenous opioid peptide involved in the control of reproductive processes in vertebrates, has also been demonstrated [31,32,36,37]. Immunohistochemistry demonstrates a seasonal variation in β-EP positivity, with a strong signal, especially in the interstitial cells of sexually quiescent lizards (December) [31].

In another study, it was shown that an additional molecule such as vitamin A and its main biologically active derivative, retinoic acid (RA), is involved in the control of lizard spermatogenesis [33]. Indeed, quantitative PCR analysis showed a different expression of its receptors (*RAR α* and *RAR β*) during the different phases of the reproductive cycle. In the reproductive period (May), only *RAR β* is expressed, while in the post-reproductive phase (August) only the transcript for *RAR α* is present [38].

More recently, through biochemical and molecular investigations in the testis of *P. siculus*, the expression of the *Vasa* gene, which encodes a protein involved in different aspects of germ cell development in many animals [39], has been demonstrated in three significant moments of the reproductive cycle: reproductive period, summer stasis, and autumn recovery [40,41]. Using immunohistochemical investigations, the authors also demonstrated the presence of the Vasa protein in germ cells, from spermatogonia to spermatids, but not in spermatozoa [41].

A major role in the control of *P. siculus* spermatogenesis is also played by steroidogenic enzymes, which represent the key to managing the local action of sex hormones on spermatogenesis, whose localization and levels have been studied (StAR, 3β-HSD, 17β-HSD, P450 aromatase, and 5α-Red) [28,35]. The presence and localization of these enzymes in the *P. siculus* testis throughout the reproductive cycle have been established by immunocytochemistry; this analysis showed that, in lizard testis, as occurs in mammals [42,43,44], sex hormones are produced by both germ cells and somatic cells [28,35].

The balance between cytochrome P450 aromatase expression and sex hormone levels is really interesting: the highest levels of P450 aromatase, the enzyme responsible for estrogen biosynthesis, and 17β-estradiol were recorded during the summer and winter stasis when the hormone is involved in blocking the spermatogenesis [15,28,45]; conversely, the highest level of testosterone and the concurrently low level of P450 aromatase were detected during the recovery period and, in particular, the reproductive period, when testosterone is responsible for the massive production of spermatozoa and the development and maintenance of secondary sexual characteristics [19,28,45].

## 4. Impairment of Spermatogenesis

Dysregulation of the factors controlling spermatogenesis severely impairs sperm production and, consequently, reproductive success. The studies carried out on *P. siculus* have played an important role in the field of reproductive toxicology, as they have led to the identification of many toxic substances capable of altering spermatogenesis, also clarifying the molecular mechanisms underlying the toxic effect in many cases.

It has been demonstrated that the balance and cooperation between the factors controlling spermatogenesis can be severely compromised by overexposure to the same physiological factors, as well as by many environmental molecules, such as herbicides and pesticides, xenoestrogens, and endocrine disruptors (EDCs).

### 4.1. Imbalance of Physiological Factors That Control Spermatogenesis

The spermatogenesis of *P. siculus* can be modified by the administration of molecules of endogenous nature, with a consequent alteration of their levels in the testis; obviously, any substance capable of modifying the concentration of these factors in the organism has a spermatogenesis effect.

In vitro studies carried out on testis organ cultures have demonstrated that the addition of both PACAP and VIP to the culture medium causes the overproduction of sex hormones such as testosterone and 17 β-estradiol; consequently, it has been hypothesized that these local factors may cooperate with each other and act on the transcription of enzymes involved in the production of hormones such as P450 aromatase. This hypothesis is supported by the fact that the gene encoding for this enzyme has a response element to the second messengers released upon the interaction of neuropeptides with their receptors [20,26,27,28,34,35]. A similar situation has been registered for D-aspartic acid (D-Asp), an endogenous amino acid that has been found in the neuroendocrine tissues of invertebrates and vertebrates [46]. In *P. siculus*, a high constitutive level of D-Asp was found in the testis, showing cyclical variations in quantity during the annual reproductive cycle in parallel with sex hormone levels. Administration of exogenous D-Asp showed that the lizard testis was specifically able to take up and accumulate this amino acid, leading to increased testosterone levels in both the testis and plasma. Exogenous D-Asp also resulted in a significant increase in mitotic activity of the testis by inducing spermatogenesis, recognized by an intense immunoreactivity of the germinal epithelium (spermatogonia and spermatids) for the proliferation cell nuclear antigen (PCNA) [29]. Differently to D-Asp, the administration of another endogenous factor such as beta-endorphin (β-EP), an opioid peptide whose localization in *P. siculus* testis has been well documented [31], has been shown to reduce the release of testosterone by inhibiting the production of pituitary gonadotropins [32].

Another important player in spermatogenesis is the steroid hormone estrogen, resulting from the aromatization of testosterone. Although estrogen is generally recognized as a female sex hormone, numerous studies demonstrate that it plays a fundamental role in controlling spermatogenesis [43]. The ability of the testis to synthesize both estrogens and estrogen receptors has been widely recognized; the widespread expression and synthesis of aromatase and estrogen receptors in the seminiferous tubules of *P. siculus* suggested a role for estrogens in modulating spermatogenesis in this organism as well, and the observation that the inhibition of P450 aromatase induced by fadrozole treatment impairs lizard spermatogenesis confirms this hypothesis [47]. In particular, endogenous estrogens in *P. siculus* males regulate spermatogenesis by blocking it during testicular quiescence and eliminating supernumerary germ cells through apoptosis during the reproductive phase [20,48].

However, as with other endogenous substances, overexposure to estrogen, or to estrogen-like substances, can induce harmful effects, with consequent alteration of the regulation of the spermatogenic process. For example, it has been demonstrated that 2 days of exposure to exogenous estradiol caused an increase in spermatogonial cell proliferation through activation of the ERK1/2 signaling pathway [49]. The prolonged treatment of 8 weeks increased the connective tissue surrounding the seminiferous tubules, whose epithelium was thin and with evident gaps between the germ cells, whereby the seminiferous tubules of the testis of the reproductive period take on a typical conformation of the stasis phase following the treatment with estrogen. This treatment also induced the expression and synthesis of vitellogenin (VTG) in males [50].

VTG, the major precursor of yolk proteins, is an estrogen-dependent and sex-specific protein synthesized in the liver of the females of oviparous vertebrates during the reproductive period [51]. In males, the protein is absent, but the *VTG* gene can be activated by exposure to estrogen or estrogen-like substances [52]. For this reason, the finding of VTG in the liver and/or plasma of oviparous males is considered a good biomarker of xeno-estrogenic pollution; however, the reason for this unusual VTG expression and synthesis has not yet been clarified.

Other data that emphasize the importance of a correct balance of estrogen during the male reproductive cycle of *P. siculus* were obtained using anti-estrogenic or estrogenic substances, such as tamoxifen or clomiphene. The first can reduce the Il-mediated increase in mast cell number in the testis and in the Harderian gland [53]. Clomiphene, a member of the selective estrogen receptor modulator (SERM) family, acts as an estrogen agonist when the hormone is in low concentration and as an antagonist at high estrogen concentrations [54]. The exposure of lizards to clomiphene during the mating period, when the estrogen level is low, led to the slowdown in spermatogenesis, the thinning of the seminiferous epithelium with degenerate germ cells, and the reduction of the secretive activity of the epididymal corpus; these changes represent the classical alterations due to estrogenic exposure [55]. Clomiphene administration to male lizards treated with FSH induces an increase in estrogen and a consequent modification in the testicular morphology. This result demonstrates that clomiphene restored the right estrogen level typical of the reproductive period [55]. All these data once again emphasize the importance of estrogen, i.e., the main female hormone, for the correct progression of spermatogenesis.

### 4.2. Effects of Pesticides and Herbicides on Spermatogenesis

The rapid growth in the use of pesticides and herbicides for agricultural production around the world has prompted many researchers to study the effects of these substances on *P. siculus* lizards, a wildlife animal living in fields and, therefore, potentially exposed to them. In a study performed in 2008 [56], it was shown that diuron, a substituted urea herbicide with a potent inhibitory effect on photosynthesis, alters male gametogenesis, causing a progressive and significant decrease in gonadosomatic index, in seminiferous tubule diameter, in spermatogonia count, in meiotic germ cells number, and the hypertrophy of the interstitial connective tissue with the recruitment of numerous lymphocytes, neutrophils, and monocytes. The authors suggest that the decrease and/or loss of germ cells could be caused by a process of necrosis leading to testicular inflammation. Furthermore, they found that diuron impaired plasma levels of sex hormones, particularly testosterone, whose values were significantly reduced in the presence of diuron, compared to 17 β-estradiol, which did not appear to be affected by the herbicide [56].

Cardone in 2012 showed that another substance, the fungicide methyl-thiophanate (MT), altered the spermatogenesis of *P. siculus*, causing sterility. Indeed, the morphological analysis demonstrated that acute treatment with this substance induced a massive reduction of germ cells in all stages of differentiation, the appearance of multinucleated giant cells, and the reduction of the lumen of the seminiferous tubules. Chronic treatment was even more harmful, inducing a significant decrease in germ cells with the appearance of vacuoles in the seminiferous epithelium and the disappearance of the lumen. In addition, the molecular analysis demonstrated a dose- and time-dependent decrease in the expression of androgen and estrogen receptors following treatment with MT [57].

Another chemical compound that has similar effects on lizard spermatogenesis is the insecticide imidacloprid. Acute and chronic exposures have shown that this substance causes a reduction of the spermatogenesis process together with alterations in plasma levels of sex hormones and in the intratesticular expression of mRNAs for androgen and estrogen receptors [58].

More recently, it has been demonstrated that the herbicide glyphosate has negative effects on *P. siculus* spermatogenesis at both molecular and histological levels [59]. In detail, oral exposure to glyphosate alters the expression of estrogen receptors in a dose-dependent manner, in particular by increasing the level of estrogen receptors’ transcripts, especially *ERβ*.

**Figure 3:**
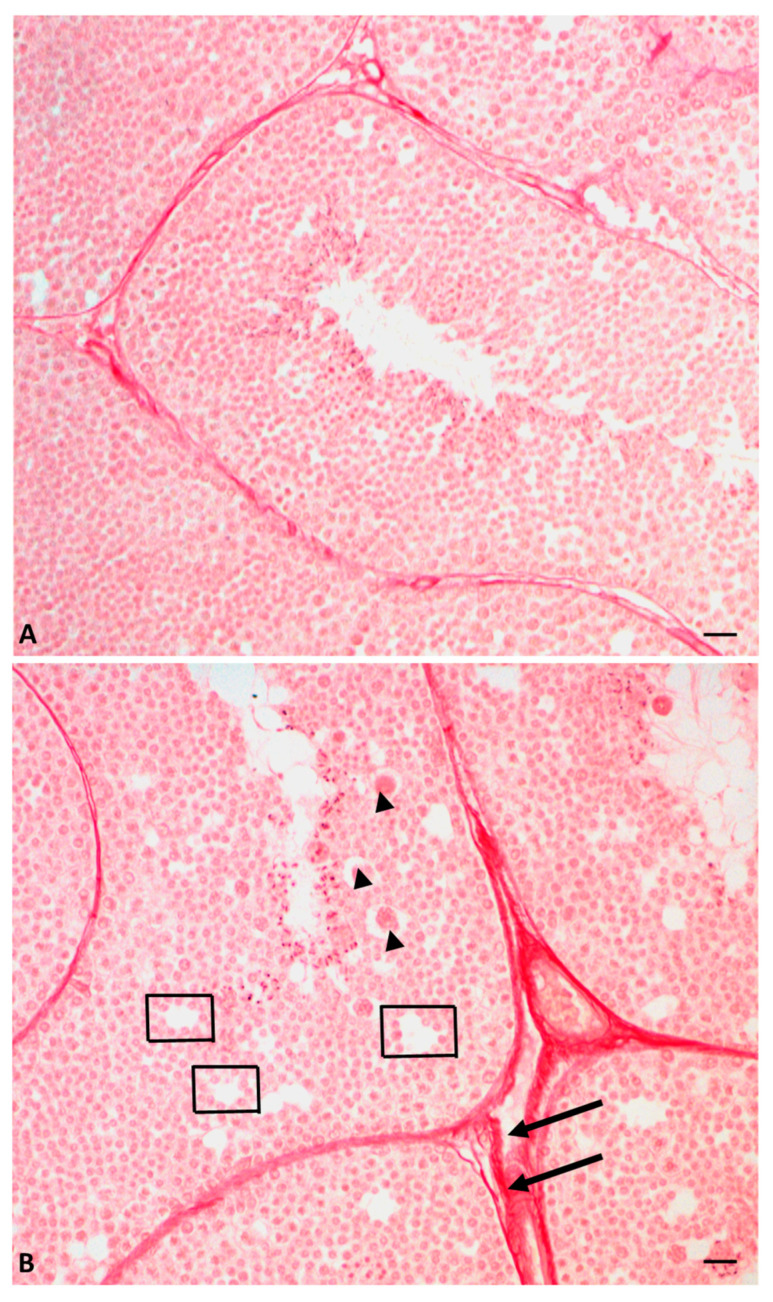
Cross-section of lizard testis during reproductive period, stained with picrosirius red: testis of control animals (**A**) and testis of glyphosate-treated animals (**B**). (**B**) Gaps (rectangles) are evident in the testicular epithelium as well as fibrosis (arrow) on the outside of the tubules. Rosette-shaped cell aggregates (arrowhead). Scale bars at 20 µm.

Immunocytochemical analysis showed that the alteration of the estrogen/estrogen receptor system causes an alteration in the structure of the seminiferous tubules by changing the distribution of connexin 43, a testis-specific gap junction protein that connects Sertoli cells and germ cells, the expression of which is regulated by estrogen. The absence of connexin 43 results in a disruption of the seminiferous epithelium, characterized by the presence of holes between the germ cells that aggregate forming structures termed rosettes. The authors also demonstrated that glyphosate induces the deposition of connective tissue between the seminiferous tubules, leading to fibrosis [59] (Figure 3), a process observed also in the liver of the same lizards exposed to glyphosate [60].

### 4.3. Endocrine Disrupting Compounds (EDCs)

Endocrine disrupting compounds are chemicals that mimic, block, or interfere with hormone activity. They are associated with various dysregulations of the body’s endocrine system, causing adverse effects in cells and tissues [61,62,63]. Given the importance of endocrine regulation in gametogenesis, it is not surprising that spermatogenesis can be altered by these substances. The routes of exposure can be multiple, as well as the origin of these compounds (from plastics, detergents, surfactants) [64].

A first study dated 1995 demonstrated that, in *P. siculus,* the testicular secretion of testosterone could be inhibited by the alkylating sulfonic ester ethane 1,2-dimethane sulfonate (EDS). Research carried out during two different moments of the reproductive cycle (winter stasis and breeding period) showed that exposure to EDS caused a reduction in androgen levels due to the impairment of Leydig cells, the main source of testosterone in the testis [65].

The effect of nonylphenol (NP), an endocrine disruptor with estrogen-like properties, was also investigated. In a first series of experiments, NP was administered to *P. siculus* via food and water and was shown to cause a slowdown in spermatogenesis through a striking structural alteration of the testis and epididymis, making them similar to those of the non-reproductive period. NP treatment has also been shown to inhibit the expression of *AR*, *ERα*, and *ERβ* genes in spermatogonia and primary spermatocytes and to shut down the secretory activity of the epididymal corpus by inducing *ERα* expression [66].

**Figure 4:**
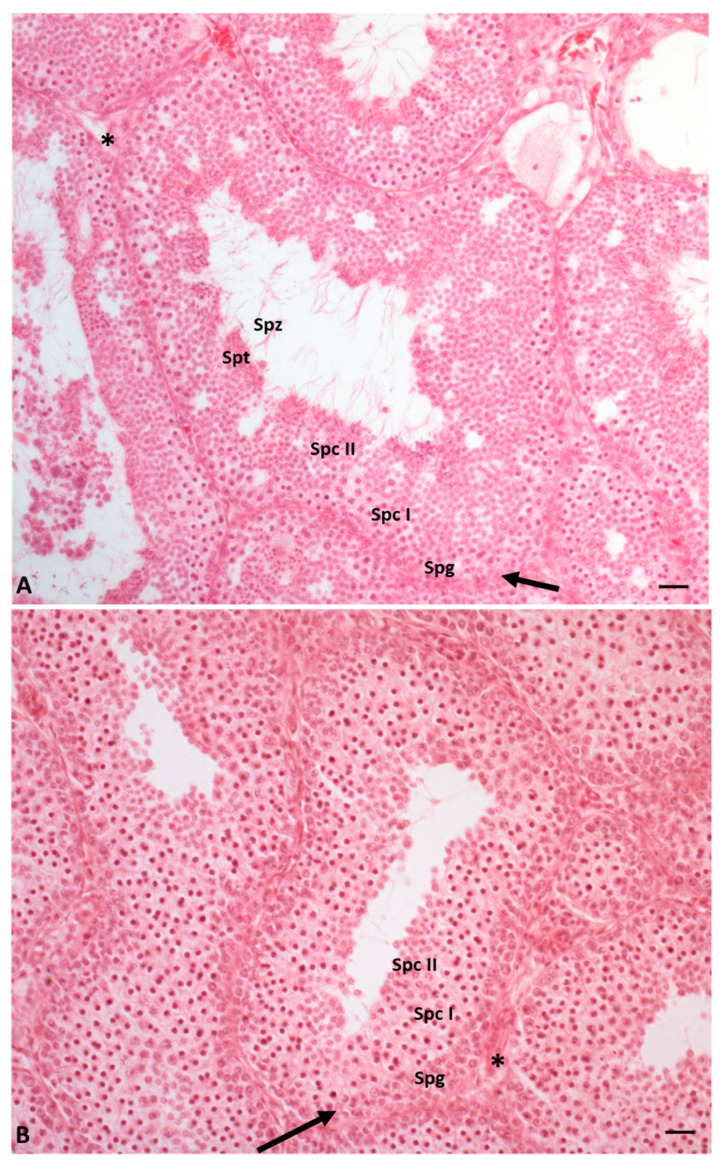
Cross-section of the lizard testis during reproductive period, stained with eosin: testis of control animals (**A**) and testis of EDCs-treated animals (**B**). (**A**) the germinal epithelium is characterized by cells in all stages of differentiation as spermatogonia (Spg), spermatocytes I and II (Spc I and II), spermatids (Spt), and spermatozoa (Spz). (**B**) The germinal epithelium is characterized by cells in the early stages of differentiation as spermatogonia (Spg), spermatocytes I and II (Spc I and II). Both spermatids (Spt) and spermatozoa (Spz) are not evident in treated animals. Sertoli cells (arrow). Leydig cells (*). Scale bars at 20 µm.

More recently, the effects of nonylphenol alone and in mixtures with another endocrine disruptor, octylphenol, on the reproductive capacity of *P. siculus* have been evaluated [67]. The data obtained from this study showed that both compounds cause a delay in spermatogenesis in a dose-dependent manner, with octylphenol prevailing over nonylphenol. In particular, the authors demonstrated that octylphenol and nonylphenol interfere with the distribution of steroidogenic enzymes, especially P450 aromatase, a key enzyme involved in the control of the different moments of spermatogenesis. The authors also showed that the treated animals had smaller seminiferous tubules with a narrowed lumen and the absence of germ cells in the last stages of differentiation such as spermatids and spermatozoa; the effects were most noticeable after exposure to octylphenol. Finally, exposure to both EDCs, alone or in combination, resulted in an increase in the presence of connective tissue outside the seminiferous tubules; this evidence, together with the data previously described, led the authors to hypothesize a delay in spermatogenesis, especially in the process of spermiogenesis [67] (Figure 4).

## 5. Conclusions

In conclusion, the data collected here show that the exogenous administration of endogenous substances that control spermatogenesis, such as neuropeptides, amino acids, and hormones, as well as any exogenous substance capable of dysregulating their production, leads to structural and functional changes in the testis. A more deleterious effect on the morphology and functionality of the seminiferous tubules was recorded following the administration of substances that directly mimic endogenous hormones such as EDCs, insecticides, and herbicides.

In particular, the changes concern the increase/decrease in the levels of estrogen, testosterone, androgen and estrogen receptors, the inhibition of the aromatase enzyme, the reduction in the levels of pituitary gonadotropins, as well as the thinning of the epithelium of the seminiferous tubules, and the loss of connections between germ cells and Sertoli cells. Table 1 lists the substances tested with the relative biomolecular effects found on the *P. siculus* spermatogenesis.

Interestingly, the morphological analysis also showed that the greatest damage found in the seminiferous tubules of differently treated males was in the reduced number of spermatids and spermatozoa, thus demonstrating that the latter are the most sensitive stages of germ cell differentiation. While this result indicates that the exogenous substances, in addition to altered levels of endogenous ones, lead to infertility due to lack of spermatozoa, on the other hand, it demonstrates that the interruption of these perturbative effects could restore the efficiency of the seminiferous tubules and the production of spermatozoa, since they do not irreversibly affect spermatogonial stem cells.

Hence, the results allow us to predict the loss of reproductive capacity of these animals, as their testis is very sensitive to the action of environmental contaminants. Sharing testicular morphology and spermatogenic activity with many other terrestrial vertebrates, including mammals, these results are also predictive of any damage to the reproductive health of all species exposed to these substances. This once again demonstrates the lizard’s ability to be an excellent but unconventional biological model.

## Figures and Tables

**Table 1:** Molecular effects of different compounds on *Podarcis siculus* spermatogenesis.

Compound	Effects	References
Neuropeptides	Increase in hormone sex levels (testosterone and 17β-estradiol).	[20,27,28]
D-aspartate	Increase in testosterone levels.	[29,30,46]
β-endorphin	Reduction of testosterone levels; reduction of pituitary gonadotropin levels.	[31,32,36,37]
Fadrozole	Inhibition of P450 aromatase.	[47]
Tamoxifene	Reduction of mast cell number in the testis and in the Harderian gland.	[53]
Clomiphene	Increase in 17β-estradiol levels; reduction of the thickness of the seminiferous epithelium; reduction of the secretive activity of the epididymal corpus.	[54,55]
17 β-estradiol	Reduction of the thickness of the seminiferous epithelium; increase in connective tissue outside the tubules; production of hepatic VTG.	[49,50,52]
Diuron	Reduction of somatic index; reduction of tubule diameter; reduction of spermatogonia meiotic cells number; hypertrophy of interstitial connective tissue; testicular inflammation; reduction of testosterone levels.	[56]
Methylthiophanate	Decrease in the expression of androgen and estrogen receptors; reduction of germ cells in all stages of differentiation; reduction of the lumen of the seminiferous tubules.	[57]
Imidacloprid	Block of spermatogenesis process; reduction of hormone sex levels (testosterone and 17β-estradiol); alteration in the expression of androgen and estrogen receptors.	[58]
Glyphosate	Increase in estrogen receptor levels; alteration of Connexin 43 distribution; fibrosis in the extratubular spaces.	[59]
EDS	Reduction of testosterone levels.	[65]
Nonylphenol	Structural alteration of the testis and epididymis; inhibition of androgen and estrogen receptor expression in germ cells; alteration of steroidogenic enzyme distribution; increase in connective tissue in the interstitial space.	[66,67]
Octylphenol	Structural alteration of the testis and epididymis; alteration of steroidogenic enzyme distribution; increase in connective tissue in the interstitial space.	[67]

## Data Availability

All data analysed during this study are included in this article.
